# Changes in Psychological Anxiety and Physiological Stress Hormones in Korea National Shooters

**DOI:** 10.3390/brainsci10120926

**Published:** 2020-12-01

**Authors:** Sang-Hyuk Park, In-Hye Park, Seung-Taek Lim, Eunjae Lee

**Affiliations:** 1 Department of Sport Science, Korea Institute of Sport Science (KISS), Seoul 01794, Korea; sang4@kspo.or.kr; 2 Department of Physical Education, Kwang Woon University, Seoul 01890, Korea; katie9910@naver.com; 3 Institute of Sport Science, Kangwon National University, Gangwon-do 24341, Korea; 4 Waseda Institute for Sport Sciences, Waseda University, Saitama 341-0018, Japan; 5 Nasaret International Hospital, Incheon 21972, Korea; 6 Center for Sport Science in Incheon, Incheon 22234, Korea

**Keywords:** stress, cortisol, salivary IgA, anxiety, shooting, athletes

## Abstract

Psychological anxiety and physiological stress hormone management is closely related to an athlete’s performance, especially in shooting competitions. Thus, we aimed to investigate the changes in saliva stress hormones according to anxiety scores of Korean elite shooters immediately before a shooting competition. Seventy-two Korean national shooting athletes (Rifle = 62, Pistol = 8, Shotgun = 2) were recruited for the present study. The physiological stress hormones were assessed based on cortisol and immunoglobulin A level in saliva. The psychological stress was assessed based on Beck Anxiety Inventory (BAI) questionnaire. Cortisol concentration and cortisol secretion rate were significant higher in severe anxiety group. Secretory immunoglobulin A (SIgA) concentration and SIgA secretion rate did not significant different in among the groups. A positive correlation was found between BAI score and cortisol concentration. These findings provide preliminary evidence indicating that psychological anxiety affects physiological stress and therefore may have a negative effect on athletes’ performance. Thus, research is needed to develop a strategy to reduce physiological stress in these athletes.

## 1. Introduction

Shooting sports athletes are assumed to be sensitive to psychological tension and anxiety pressure [1]. This sport’s athletes need attentional control to focus on skill execution and prevent distracting thoughts from impairing performance [2]. The attention control theory focuses on explaining the psychological changes of athletes related to anxiety and cognitive performance. The theory is that the effects of anxiety on attention control are key to understanding the relationship between anxiety and performance. Generally, the anxiety experienced by shooters in the competition has a negative effect on the attention control to the competition, while compromising psychological stability. Attention control function might be impaired with higher anxiety, this process can negatively affect attention and concentration. Therefore, psychological and physiological control is important for improving shooting performance.

The Beck Anxiety Inventory (BAI) is widely used as a prominent screening and outcome research tool for measuring psychological anxiety [3]. Two factors have been identified for the BAI: somatic and subjective anxiety/panic based on the work of Beck et al. [4]. The 21-item self-report inventory was designed to assess the severity of anxiety symptoms in adults and adolescents [5]. A previous study reported that 150 runners’ BAI anxiety scores were positively correlated with distress [6], and a significant negative relationship was also demonstrated between the athletes’ competitive anxiety levels and their athletic experiences [7].

In addition, higher BAI scores are associated with impaired physical performance [8]. This is why mental and emotional states (i.e., anxiety states) directly affect the autonomic nervous system [9]. Stress is defined as a state of threatened homeostasis following exposure to extrinsic or intrinsic adverse forces [10]. Continuing and excessive stress causes instability in the body’s homeostasis, cardiovascular system, musculoskeletal response, emotional stability, and immune function [11]. In physiological terms two components physiological markers of stress—salivary cortisol and secretory immunoglobulin A (SIgA)—have been proposed [12,13,14].

Previous studies reported that salivary cortisol and SIgA concentrations were significantly elevated immediately after a competitive 5000-m race [15]. The SIgA concentrations in volleyball players were significantly lower than those in sedentary controls, and the salivary cortisol concentrations in volleyball players were markedly higher than those in sedentary controls [16]. Cortisol concentrations in college male athletes were significantly higher with increasing training intensity, however as the increasing training intensity, the SIgA was significantly decreased [17].

Therefore, psychological and physiological management is closely related to an athlete’s performance, especially in shooting competitions. However, research on the psychological anxiety and physiological stress of athletes in shooting sports is lacking. Thus, we aimed to investigate the changes in psychological anxiety and physiological stress hormones in Korean elite shooters immediately before a shooting competition.

## 2. Material and Methods

### 2.1. Participants

Seventy-two Korean national shooting athletes (Rifle = 62, Pistol = 8, Shotgun = 2) were recruited for the present study. The following criteria excluded athletes from participating in the saliva-based stress tests: (1) heart, lung, or metabolic disorders, (2) diagnosed with major depression and bipolar disorder, and schizophrenia (Diagnostic and Statistical Manual of Mental Disorders: DSM-IV) within the past year, (3) diagnosed with mental symptoms such as anxiety and agitation within the past three months, and (4) diagnosed with a musculoskeletal disorder that precluded safe participation in shooting competitions.

All participants were screened for anxiety scale using the Beck Anxiety Inventory (BAI) [4]. According to a baseline BAI cutoff score, participants were divided into four groups, including the no anxiety group (scores: 0–9 points), mild anxiety group (scores: 10–19 points), moderate anxiety group (scores: 20–29 points) and severe anxiety group (scores < 29 points): mild anxiety group (*n* = 12, Rifle = 10, Pistol = 2, Shotgun = 0), moderate anxiety group (*n* = 21, Rifle = 20, Pistol = 1, Shotgun = 0), and severe anxiety group (*n* = 39, Rifle = 32, Pistol = 5, Shotgun = 2).

All subjects who agreed to participate in the study had the study explained to them to ensure a complete understanding of its purpose and the methods used with the ethical standard of the Declaration of Helsinki. The subjects also signed an informed consent form before participation. This study was approved by This study was approved by the Institutional Review Board at the Korea Institute of Sport Science, Seoul, South Korea (KISS-1906-016-01).

The characteristics of the participants are shown in Table 1.

### 2.2. Beck Anxiety Inventory (BAI) Questionnaire

Subjects completed the BAI, which is a 21-item self-report inventory for assessing the severity of clinical anxiety [4]. Subjects rated each item on a 4-point scale ranging from 0 (“Not at all”) to 3 (“I could barely stand it”) with regard to their anxiety-related symptoms during the past week. The BAI is scored by adding the severity ratings across all 21 items; total scores can range from 0 to 63. Scores were classified as follows: 0–9, no anxiety; 10–19, mild anxiety; 20–29, moderate anxiety; and over 30, severe anxiety. Internal consistency and test-retest reliability of the Korean BAI have been reported as 0.91–0.93 and r = 0.84, respectively [18,19].

### 2.3. Saliva Collection

Saliva samples were collected using the same method used in an earlier study [20]. Participants sat and rinsed their mouths three times with distilled water for 30 s and then rested for 5 min. Saliva production was stimulated by chewing a sterile cotton (Salivette: Sersted, Nümbrecht, Germany) at a frequency of 60 cycles per min. The obtained saliva samples were separated from the cotton by centrifugation at 3500 rpm for 10 min. We measured the saliva volume secreted by chewing for 1 min and expressed it as the saliva flow rate (mL/min). After the measurement of the sample volume, saliva samples were frozen at −50 °C and stored until the end of the study period.

### 2.4. Stress Hormones Determination

Salivary stress hormones cortisol and SIgA were obtained immediately before the competition. Saliva volume was estimated assuming saliva density to be 1.00 g·mL^−1^ [21], and saliva flow rate was calculated using saliva volume and collection time. The salivary stress hormone concentration was determined in duplicate by a sandwich enzyme-linked immunosorbent assay (ELISA), with a within assay coefficient of variation of 2.8 ± 3.5%.

For the analysis of stress hormone levels, data were expressed in two forms: (a) absolute concentrations of cortisol and SIgA (µg/mL), and (b) stress hormone secretion rate (mL/min), or the total amount of stress hormones appearing on the mucosal surface per unit of time. The stress hormone secretion rate was calculated by multiplying the absolute stress hormone concentration (µg/mL) by the saliva flow rate (mL/min), which was calculated by dividing the total volume of saliva obtained in each sample (mL) by the time taken to produce the saliva sample (min) [22].

### 2.5. Statistical Analysis

SPSS statistical package version 25.0 for Windows (SPSS, Inc., Chicago, IL, USA) was used to perform all statistical analyses. Means and standard deviations were computed for all variables, and normality was checked using the Shapiro–Wilk test. Non-normal data were converted using square root (saliva flow rate) or logarithmic (concentration and secretion rate of stress hormones) transformations that achieved normality for all variables. The participants’ stress hormones (cortisol and SIgA) were further analyzed for significant differences among the groups using a one-way ANOVA. Pearson correlation coefficients analysis and a stepwise multiple regression analysis were conducted to examine the effect of the level of stress hormones on anxiety. Post hoc analysis (Tukey test) was used to compare specific differences when significance was found. Statistical significance was accepted at the 0.05 level.

## 3. Results

### 3.1. Cortisol According to BAI

**Figure 1:**
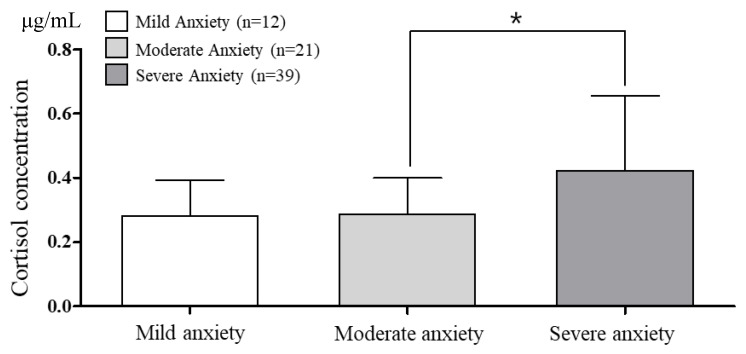
The comparison of cortisol concentration according to Beck Anxiety Inventory (BAI) anxiety. α = 0.05, * *p* = 0.011. df = 2, *p* = 0.011, et^2^ = 0.123, observed power = 0.784.

**Figure 2:**
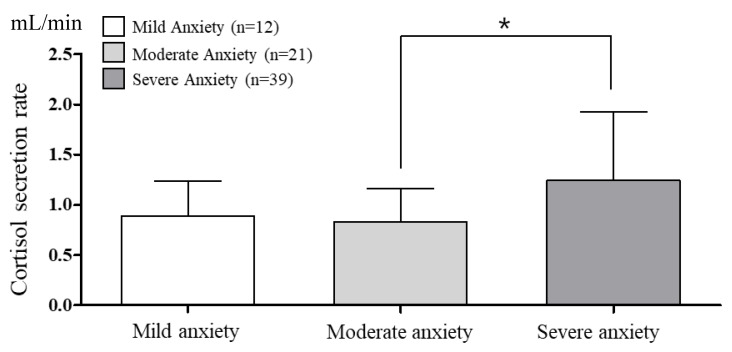
The comparison of cortisol secretion rate according to BAI anxiety. α = 0.05, * *p* = 0.014. df = 2, *p* = 0.014, et^2^ = 0.125, observed power = 0.753.

The cortisol concentrations and secretion rates according to BAI anxiety are presented in Figure 1 and Figure 2. The saliva flow rate was 3.02 ± 0.29 mL/min. One-way ANOVA revealed a significantly higher cortisol concentration in the severe anxiety group (*p* = 0.011). The cortisol secretion rate was also significantly higher in the severe anxiety group than in the moderate anxiety group (*p* = 0.014).

### 3.2. SIgA According to BAI

**Figure 3:**
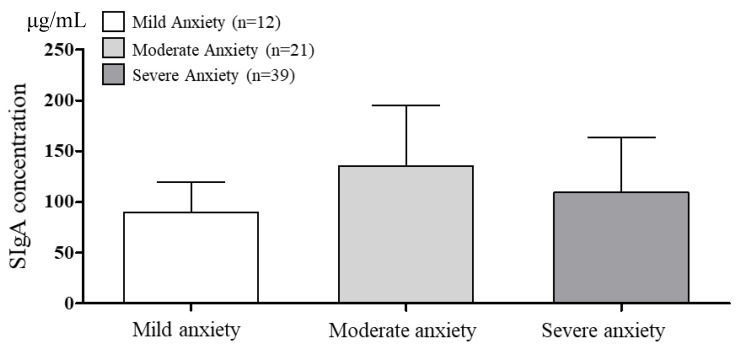
The comparison of SIgA concentration according to BAI anxiety. α = 0.05, *p* = 0.050 df = 2, *p* = 0.050 et^2^ = 0.083, observed power = 0.583.

**Figure 4:**
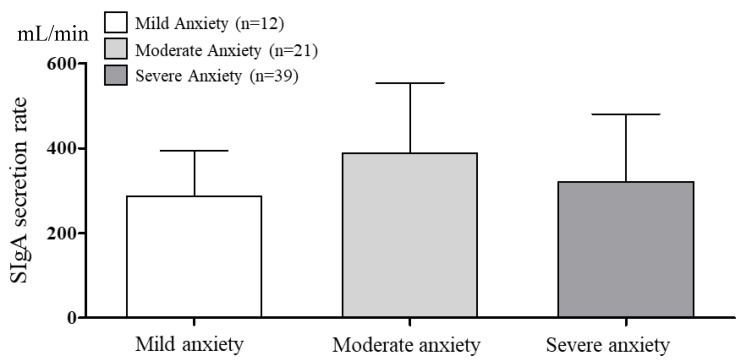
The comparison of SIgA secretion rate according to BAI anxiety. α = 0.05, *p* = 0.685 df = 2, *p* = 0.685 et^2^ = 0.011, observed power = 0.109.

The SIgA concentrations and secretion rates according to BAI anxiety are presented in Figure 3 and Figure 4. The saliva flow rate was 3.02 ± 0.29 mL/min. One-way ANOVA revealed no significant SIgA concentration among the groups (*p* = 0.050). The SIgA secretion rate was also not significant among the groups (*p* = 0.685).

### 3.3. Correlations between the BAI Score and the Stress Hormones

Table 2 shows the correlation coefficients of the BAI score and stress hormones. A positive correlation was found between the BAI score and cortisol concentration (*p* < 0.05). Positive correlations were found between concentrations and secretion rates in both stress hormones (*p* < 0.01; *p* < 0.01, respectively).

### 3.4. Multiple Regression Analysis between BAI Anxiety and Cortisol Concentration

Subsequently, a multiple regression analysis was conducted based on the above correlations. These results are presented in Table 3. As indicated in Table 3, there was a significant relationship between shooting athletes’ BAI anxiety and cortisol concentration levels (*p* < 0.05).

## 4. Discussion

The main finding of this study was that cortisol concentration levels and cortisol secretion rates were significantly higher in the severe anxiety group than in the moderate anxiety group. In addition, a positive correlation was found between BAI scores and cortisol concentrations. Thus, the findings suggest that shooting athletes’ anxiety increased levels of the stress hormone cortisol.

Statistically significant associations were also found between sport-specific psychological abilities and different measures of mood in athletes [23]. Among the most relevant psychological signs associated with sports performance was competitive state anxiety, which improved the process of adaptation to competition [24]. Causes of higher anxiety in athletes can be detrimental in performance situations [25]. Ngo et al. reported that athletes with lower levels of anxiety obtained better results in competitions, while individuals with higher levels of anxiety obtained worse results [26]. Romyn et al. showed that athletes’ anxiety increased before a 7-day training week and a 7-day competitive tournament [27]. The present study demonstrated the BAI anxiety states of athletes: mild anxiety was observed in 12 athletes, moderate anxiety in 21 athletes, and severe anxiety in 39 athletes immediately before the competition. In total, 54.17% of the athletes experienced severe anxiety immediately before a competition. Shooting competitions are individual sports that require high levels of concentration. Individual sports athletes are thus more likely to report anxiety and depression than team sports athletes [28]. Moreover, in this study, the severe anxiety group had higher cortisol levels and secretion rates than the other groups. Levels of state anxiety and trait anxiety are positively and directly related in both individual and team sports, although they are more elevated in individual sports.

Cortisol and SIgA are steroid hormones that change in response to psychological and physical stress [29]. An increase in cortisol is associated with relatively intense psychological stress [30]. Higher SIgA is associated with mental state [31]. There is a positive correlation between psychological stress and cortisol levels and a negative association between psychological stress and SIgA levels [32]. Oshima et al. reported that acute physiological training stress resulted in an increase in resting plasma cortisol concentration [17]. He et al. reported that significant decreases in secretion rates of SIgA were observed at times of intense training and competition [33]. Intensive training in combination with rapid weight changes resulted in increased SIgA and cortisol stress responses in elite female taekwondo athletes [34]. In this study, we observed significantly higher cortisol concentration levels and secretion rates in the severe anxiety group than in the moderate and mild anxiety groups. However, there was no significant difference in SIgA concentration and secretion rate levels among the groups. In humans, perceived stress activates the central nervous system (CNS), releasing corticotropin-releasing hormone (CRH) from the hypothalamus, adrenal corticotropic hormone (ACTH) in the anterior pituitary gland, and cortisol in the adrenal cortex [35]. The entire hypothalamus–pituitary–adrenal system is designed to allow organisms to adapt to physical, mental, and social changes in their environment [35]. These results suggest that immediately before competitions stress may be involved in the release of the physiological stress hormone cortisol. In addition, the positive correlations between BAI anxiety and cortisol concentration levels and multiple regression analysis revealed a significant relationship between shooting athletes’ BAI anxiety and cortisol concentration. Nevertheless, cortisol was not significant between the mild anxiety and moderate anxiety groups. Takahashi et al. reported that individuals with high degrees of perceived autonomic reactivity have higher levels of basal cortisol and perceived stress response [36]. Maybe the difference in the degree of response to stress by individuals. Although the groups were divided by anxiety, the degree of cortisol response to stress may be different.

Previous studies on SIgA were inconclusive. No changes were observed for SIgA measurements before Jiu-Jitsu matches [37]. A significant increase was observed during competition in SIgA and cortisol, and the results demonstrated that performance in major competition induces a stress response in athletes [38]. In this study, there was no significant difference in SIgA concentration and secretion rate levels among the groups; however, SIgA in the moderate anxiety group was higher than in the severe anxiety group. Birkett et al. reported that SIgA activity in response to psychosocial stressors, however SIgA’s response was different for male and female participants [39]. Unfortunately, in this study, groups were divided by anxiety and did not distinguish between male and female. It is thought that the reason why the SIgA did not respond in this study was because it did not distinguish between male and female. However, based on the elevated stress response observed, modifications to individual post-race recovery protocols may be required to enable athletes to maximize performance across all days of competition [38].

The present study has some limitations and points to suggestions for further research. We did not control for factors such as social stress and addictive activity (food, and medicine that affects stress hormones). In particular, the intake of prescription drugs plays an important role in hormonal changes in the body. We assumed that because the subjects lived fairly stable lives for several years, there would be no significant differences in social stress and addictive activities. In future studies, exercise (meditation or yoga) interventions to reduce anxiety are needed. In addition, it is necessary to find out how to exercise dynamically rather than statically to reduce anxiety. Another limitation is that the small number of subjects included could limit the statistical significance of the results, and further studies with larger populations are required to validate our findings. The last one concerns the change in SIgA. There is a pervious study in SIgA that shows different responses by gender [39], so further study of gender classification is needed.

## 5. Conclusions

In conclusion, this study examined changes in psychological anxiety and physiological stress hormones in Korean elite shooters immediately before a shooting competition. In addition, cortisol concentration levels and cortisol secretion rates were significantly higher in the severe anxiety group than in the moderate anxiety group. Moreover, positive correlations between BAI anxiety and cortisol concentration levels, and multiple regression analysis revealed a significant relationship between shooting athletes’ BAI anxiety and cortisol concentration levels.

Therefore, these findings provide preliminary evidence indicating that psychological anxiety affects physiological stress and therefore may have a negative impact on athletes’ performance. Thus, research is needed to develop a strategy to reduce physiological stress in these athletes.

## Figures and Tables

**Table 1:** The characteristic of the participants.

Variable	Mild Anxiety (*n* = 12)	Moderate Anxiety (*n* = 21)	Severe Anxiety (*n* = 39)	Total (*n* = 72)
Male (*n*)	6	9	9	24
Female (*n*)	6	12	30	48
Age (years)	22.83 ± 4.49	22.90 ± 5.15	23.72 ± 4.22	23.33 ± 4.50
Careers (years)	10.67 ± 4.25	10.52 ± 5.04	11.67 ± 4.35	11.17 ± 4.51

**Table 2:** Correlations coefficients between the BAI score and the stress hormones.

Variable	BAI Score	CC	SC	CSR	SSR
BAI score	-				
CC	0.248 *	-			
SC	0.109	0.044	-		
CSR	0.220	0.988 **	0.013	-	
SSR	0.081	0.063	0.987 **	0.040	-

BAI, beck anxiety inventory; CC, cortisol concentration; SC, SIgA concentration; CSR, cortisol secretion rate; SSR, SIgA secretion rate. *: *p* < 0.05, **: *p* < 0.01.

**Table 3:** Multiple regression analysis between BAI anxiety and cortisol concentration.

Dependent Variable	Independent Variable	R^2^	R^2^C	β	F-Value
BAI anxiety	Cortisol	0.061	0.061	−0.248	4.583 *

* *p* < 0.05.

## Abbreviations

SIgA: secretory immunoglobulin-A; BAI: beck anxiety inventory; CNS: central nervous system; CRH: corticotropin-releasing hormone; ACTH: adrenal corticotrophic hormone; SPSS: statistical package for social science; ANOVA: analysis of variance.

## References

[1] Solberg E.E., Berglund K.A., Engen O., Ekeberg O., Loeb M. (1996). The effect of meditation on shooting performance. Br. J. Sports Med..

[2] Boutcher S.H., Crews D.J. (1987). The effect of a preshot attentional routine on a well-learned skill. Int. J. Sport. Psychol..

[3] Bardhoshi G., Duncan K., Erford B.T. (2016). Psychometric Meta-Analysis of the English Version of the Beck Anxiety Inventory. J. Couns. Dev..

[4] Beck A.T., Epstein N., Brown G., Steer R.A. (1988). An inventory for measuring clinical anxiety: Psychometric properties. J. Consult. Clin. Psychol..

[5] Osman A., Barrios F.X., Gutierrez P.M., Williams J.E., Bailey J. (2008). Psychometric properties of the Beck Depression Inventory-II in nonclinical adolescent samples. J. Clin. Psychol..

[6] Wilson P.B. (2018). Perceived life stress and anxiety correlate with chronic gastrointestinal symptoms in runners. J. Sports Sci..

[7] Mottaghi M., Atarodi A., Rohani Z. (2013). The Relationship between Coaches’ and Athletes&rsquo; Competitive Anxiety, and their Performance. Iran. J. Psychiatry Behav. Sci..

[8] Zhang M., Kim J.C., Li Y., Shapiro B.B., Porszasz J., Bross R., Feroze U., Upreti R., Martin D., Kalantar-Zadeh K. (2014). Relation Between Anxiety, Depression, and Physical Activity and Performance in Maintenance Hemodialysis Patients. J. Ren. Nutr..

[9] Kayihan G., Ersöz G., Ozkan A., Koz M., Kayıhan G. (2013). Relationship between efficiency of pistol shooting and selected physical-physiological parameters of police. Int. J. Police Strat. Manag..

[10] Chrousos G.P., Gold P.W. (1992). The concepts of stress and stress system disorders. Overview of physical and behavioral homeostasis. JAMA.

[11] Song E.J., Lee M.Y. (2018). Effects of Aromatherapy on Stress Responses, Autonomic Nervous System Activity and Blood Pressure in the Patients Undergoing Coronary Angiography: A Non-Randomized Controlled Trial. J. Korean Acad. Nurs..

[12] Malhi G.S., Mann J.J. (2018). Depression. Lancet.

[13] Staley M., Conners M.G., Hall K., Miller L.J. (2018). Linking stress and immunity: Immunoglobulin A as a non-invasive physiological biomarker in animal welfare studies. Horm. Behav..

[14] Pritchard B.T., Stanton W., Lord R., Petocz P., Pepping G.-J. (2017). Factors Affecting Measurement of Salivary Cortisol and Secretory Immunoglobulin A in Field Studies of Athletes. Front. Endocrinol..

[15] Li C.-Y., Hsu G.-S., Suzuki K., Ko M.-H., Fang S.-H. (2015). Salivary Immuno Factors, Cortisol and Testosterone Responses in Athletes of a Competitive 5000 m Race. Chin. J. Physiol..

[16] Li T.L., Lin H.C., Ko M.H., Chang C.K., Fang S.H. (2012). Effects of prolonged intensive training on the resting levels of salivary immunoglobulin A and cortisol in adolescent volleyball players. J. Sports Med. Phys. Fit..

[17] Oshima S., Takehata C., Sasahara I., Lee E., Akama T., Taguchi M. (2017). Changes in Stress and Appetite Responses in Male Power-Trained Athletes during Intensive Training Camp. Nutrients.

[18] Kwon S.M., Lim Y.J. (2007). The State-Trait Anxiety Inventory, Trait Version: Examination of a Method Factor. Korean Soc. Sci. J..

[19] Lee H.-K., Hong S., Lee E.-H., Taeg H.S., Kim J. (2016). Psychometric Properties of the Beck Anxiety Inventory in the Community-dwelling Sample of Korean Adults. Korean J. Clin. Psychol..

[20] Akimoto T., Kumai Y., Akama T., Hayashi E., Murakami H., Soma R., Kuno S., Kono I. (2003). Effects of 12 months of exercise training on salivary secretory IgA levels in elderly subjects. Br. J. Sports Med..

[21] Cole A.S., Eastoe J.E. (1988). Biochemistry and Oral Biology.

[22] MacKinnon L.T., Jenkins D.G. (1993). Decreased salivary immunoglobulins after intense interval exercise before and after training. Med. Sci. Sports Exerc..

[23] Reigal R.E., Diz J.A.V., Morillo J.P., Hernández-Mendo A., Morales-Sánchez V. (2019). Psychological Profile, Competitive Anxiety, Moods and Self-Efficacy in Beach Handball Players. Int. J. Environ. Res. Public Health.

[24] Kuan G., Morris T., Kueh Y.C., Terry P.C. (2018). Effects of Relaxing and Arousing Music during Imagery Training on Dart-Throwing Performance, Physiological Arousal Indices, and Competitive State Anxiety. Front. Psychol..

[25] Patel D.R., Omar H., Terry M. (2010). Sport-related Performance Anxiety in Young Female Athletes. J. Pediatr. Adolesc. Gynecol..

[26] Ngo V., Richards H., Kondrič M. (2017). A Multidisciplinary Investigation of the Effects of Competitive State Anxiety on Serve Kinematics in Table Tennis. J. Hum. Kinet..

[27] Romyn G., Robey E., Dimmock J.A., Halson S.L., Peeling P. (2016). Sleep, anxiety and electronic device use by athletes in the training and competition environments. Eur. J. Sport Sci..

[28] Pluhar E., McCracken C., Griffith K.L., Christino M.A., Sugimoto D., Meehan W.P. (2019). Team Sport Athletes May Be Less Likely to Suffer Anxiety or Depression than Individual Sport Athletes. J. Sports. Sci. Med.

[29] Papacosta E., Nassis G.P. (2011). Saliva as a tool for monitoring steroid, peptide and immune markers in sport and exercise science. J. Sci. Med. Sport.

[30] Simons S.S., Cillessen A.H., de Weerth C. (2017). Cortisol stress responses and children’s behavioral functioning at school. Dev. Psychobiol..

[31] Yaniv A.U., Djalovski A., Priel A., Zagoory-Sharon O., Feldman R. (2018). Maternal depression alters stress and immune biomarkers in mother and child. Depress. Anxiety.

[32] Moirasgenti M., Doulougeri K., Panagopoulou E., Theodoridis T. (2019). Psychological stress reduces the immunological benefits of breast milk. Stress Health.

[33] He C.-S., Tsai M.-L., Ko M.-H., Chang C.-K., Fang S.-H. (2010). Relationships among salivary immunoglobulin A, lactoferrin and cortisol in basketball players during a basketball season. Eur. J. Appl. Physiol..

[34] Tsai M.-L., Ko M.-H., Chang C.-K., Chou K.-M., Fang S.-H. (2010). Impact of intense training and rapid weight changes on salivary parameters in elite female Taekwondo athletes. Scand. J. Med. Sci. Sports.

[35] Burke H.M., Davis M.C., Otte C., Mohr D.C. (2005). Depression and cortisol responses to psychological stress: A meta-analysis. Psychoneuroendocrinology.

[36] Takahashi T., Ikeda K., Ishikawa M., Kitamura N., Tsukasaki T., Nakama D., Kameda T. (2005). Anxiety, reactivity, and social stress-induced cortisol elevation in humans. Neuro Endocrinol. Lett..

[37] Moreira A., Franchini E., De Freitas C.G., Arruda A.F.S., De Moura N.R., Costa E.C., Aoki M.S. (2012). Salivary Cortisol and Immunoglobulin A Responses to Simulated and Official Jiu-Jitsu Matches. J. Strength Cond. Res..

[38] Sinnott-O’Connor C., Comyns T.M., Nevill A.M., Warrington G., Comyns T. (2018). Salivary Biomarkers and Training Load During Training and Competition in Paralympic Swimmers. Int. J. Sports Physiol. Perform..

[39] Birkett M., Johnson L., Gelety C. (2017). Investigation of Sex Differences In sIgA Response to the Trier Social Stress Test. Stress Health.

